# Supplementary material for: The Recretohalophyte Tamarix TrSOS1 Gene Confers Enhanced Salt Tolerance to Transgenic Hairy Root Composite Cotton Seedlings Exhibiting Virus-Induced Gene Silencing of GhSOS1
Source: Int J Mol Sci. 2019 Jun 15;20(12):2930. doi: 10.3390/ijms20122930 (PMC6628528; doi:10.3390/ijms20122930)
Supplement: Supplementary file 1 [file ijms-20-02930-s001.pdf]

Supplementary Materials:

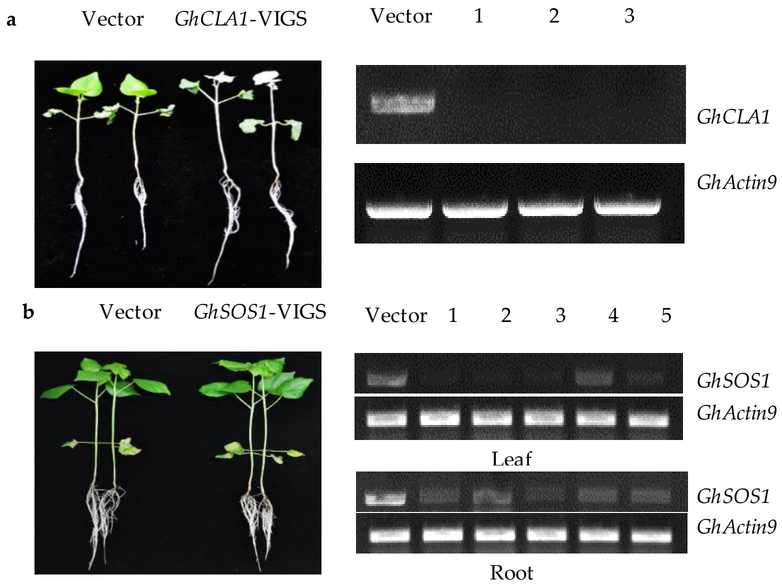

Figure S1. Construction and identification of *GhCLA1*-VIGS or *GhSOS1*-VIGS cotton plants. **(a)** *GhCLA1*-VIGS cotton plants infected with *Agrobacterium tumefaciens* GV3101 containing pTRV1 and pTRV2-*GhCLA1* showed photobleaching phenotypes, and the empty-vector-transformed plants infected with *A. tumefaciens* GV3101 containing pTRV1 and pTRV2 was as a control (**left**); after 10 days of infection, the gene expression patterns of *GhCLA1* in the leaves of Vector and *GhCLA1*-VIGS plants was analyzed by semi-quantitative RT-PCR, and the housekeeping gene *GhActin9* was used as an internal control (right). **(b)** *GhSOS1*-VIGS cotton plants infected with *A. tumefaciens* GV3101 containing pTRV1 and pTRV2-*GhSOS1* and empty-vector-transformed plants was cultured with 1/2 Hoagland solution (**left**), the gene expression patterns of *GhSOS1* in the roots and leaves of Vector and *GhSOS1*-VIGS plants was analyzed by semi-quantitative RT-PCR (right).

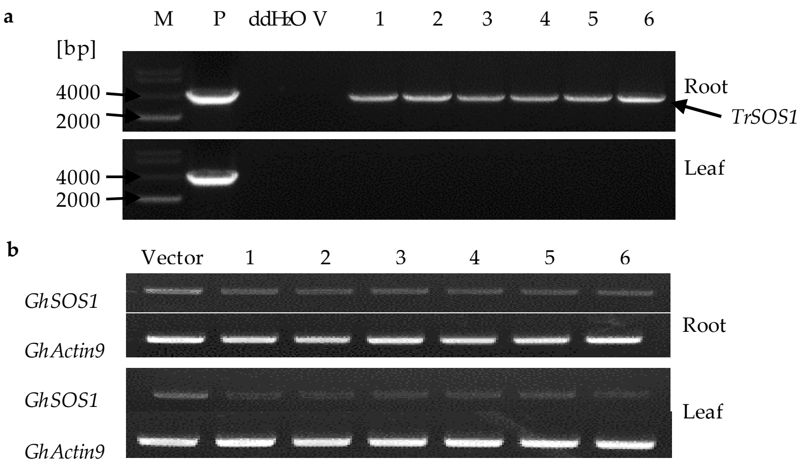

Figure S2. Construction and identification of hairy root composite cotton plants of *hrGhSOS1*-VIGS or *hrTrSOS1*-OE/*GhSOS1*-VIGS. **(a)** The plants were firstly infected with pSuper+1300-*TrSOS1*-containing *Agrobacterium rhizogenes* strain K599 to obtain *TrSOS1*-overexpressed hairy root composite cotton plants (*hrTrSOS1*-OE), the gene expression patterns of *TrSOS1* in the roots and leaves was analyzed using DNA as the template by PCR. M: DNA marker; P: Positive control; V: Empty vector, represented as the negative control; ddH$_2$O: Distilled deionized water, also represented as the negative control; **(b)** The abovementioned empty-vector- or *TrSOS1*-transformed cotton plants were infected with *A. tumefaciens* GV3101 containing pTRV1 and pTRV2-*GhSOS1* to obtain *hrGhSOS1*-VIGS or *hrTrSOS1*-OE/*GhSOS1*-VIGS plants. The plants infected with *A. tumefaciens* GV3101 containing pTRV1 and pTRV2 was used as a control (Vector), the gene expression patterns of *GhSOS1* in the roots and leaves of *hrGhSOS1*-VIGS or *hrTrSOS1*-OE/*GhSOS1*-VIGS plants were analyzed by semi-quantitative RT-PCR.
